# The Neuromodulation of the Intestinal Immune System and Its Relevance in Inflammatory Bowel Disease

**DOI:** 10.3389/fimmu.2015.00590

**Published:** 2015-11-20

**Authors:** Martina Di Giovangiulio, Simon Verheijden, Goele Bosmans, Nathalie Stakenborg, Guy E. Boeckxstaens, Gianluca Matteoli

**Affiliations:** ^1^Department of Clinical and Experimental Medicine, Translational Research Center for Gastrointestinal Disorders (TARGID), KU Leuven, Leuven, Belgium

**Keywords:** intestinal immune system, oral tolerance, sympathetic system, parasympathetic system, peptidergic pathway, neuropeptide, inflammatory bowel disease

## Abstract

One of the main tasks of the immune system is to discriminate and appropriately react to “danger” or “non-danger” signals. This is crucial in the gastrointestinal tract, where the immune system is confronted with a myriad of food antigens and symbiotic microflora that are in constant contact with the mucosa, in addition to any potential pathogens. This large number of antigens and commensal microflora, which are essential for providing vital nutrients, must be tolerated by the intestinal immune system to prevent aberrant inflammation. Hence, the balance between immune activation versus tolerance should be tightly regulated to maintain intestinal homeostasis and to prevent immune activation indiscriminately against all luminal antigens. Loss of this delicate equilibrium can lead to chronic activation of the intestinal immune response resulting in intestinal disorders, such as inflammatory bowel diseases (IBD). In order to maintain homeostasis, the immune system has evolved diverse regulatory strategies including additional non-immunological actors able to control the immune response. Accumulating evidence strongly indicates a bidirectional link between the two systems in which the brain modulates the immune response via the detection of circulating cytokines and via direct afferent input from sensory fibers and from enteric neurons. In the current review, we will highlight the most recent findings regarding the cross-talk between the nervous system and the mucosal immune system and will discuss the potential use of these neuronal circuits and neuromediators as novel therapeutic tools to reestablish immune tolerance and treat intestinal chronic inflammation.

## Intestinal Immune Homeostasis and Oral Tolerance

A main function of the immune system is to distinguish between “danger” or “non-danger” signals and to respond appropriately. This is crucial in the gastrointestinal (GI) tract, where the immune system is constantly exposed to a multitude of food antigens and symbiotic microflora, which are essential for providing vital nutrients to the body. Therefore, the balance between immune activation versus tolerance should be tightly regulated to maintain intestinal homeostasis. In recent years, it has become clear that the mucosal immune system has developed an ingenious mechanism, referred to as oral tolerance, to fulfill this task. In detail, *lamina propria* antigen-presenting cells (APCs), such as dendritic cells (DCs) and macrophages (MFs), are “educated” by intestinal bioactive factors, such as transforming growth factor-beta (TGF-β), retinoic acid, thymic stromal lymphopoietin, and mucins (1–5), to suppress inflammation and promote immunological tolerance via the induction and expansion of antigen specific anti-inflammatory regulatory T cells (Tregs) in the mesenteric lymph nodes (MLN).

To date, oral tolerance is widely accepted to represent the cornerstone of intestinal immune homeostasis (3, 6). In healthy individuals, intestinal immune tolerance against food and microbiota antigens is, therefore, crucial to prevent an immune reaction against harmless food (3). Nevertheless, in individuals with genetic or environmental predisposition (altered microbiota, viral or bacterial infection, chemical additives, or pollution) oral tolerance is broken resulting in immune activation against luminal antigens. Loss of this delicate equilibrium indiscriminately results in chronic and excessive immune activation indiscriminately against luminal antigens leading to invalidating intestinal disorders, such as inflammatory bowel disease (IBD). So far, there is no cure for IBD. The actual goal of IBD treatment is to reduce the inflammation that triggers symptoms and tissue alterations. In a group of cases, this may lead to long-term remission and reduced risks of complications but in a large number of patients disease relapses are common. Thus, the ultimate aim in IBD research is to explore novel therapeutic methods to reinstall intestinal immune tolerance.

Lately, experimental and clinical evidence suggests that an additional actor, i.e., the nervous system, may play a critical role in modulating the intestinal microenvironment, preserving immune homeostasis and tolerance. In the current review, we will highlight the most recent findings regarding the cross-talk between the nervous system and the mucosal immune system. Furthermore, we will discuss the potential employment of some of these neuronal circuits and neuromediators as novel therapeutic tools to reestablish immune tolerance and treat intestinal chronic inflammatory diseases, such as IBD.

## The Nervous System as Modulator of Immune Response

The cross-talk between the immune and nervous systems occurs through a complex set of neurotransmitters, cytokines and hormones and is undoubtedly playing a crucial role in the regulation of an immune response (7). The ground-breaking idea that neurotransmitters could serve as immune modulators emerged with the discovery that their release and diffusion from nervous tissue could lead to signaling through typical neurotransmitter receptors expressed on immune cells (8).

Inflammatory mediators released locally can activate sensory nerves and send signals to the nervous system. Through the induction of the so called “inflammatory reflex,” efferent nerves also convey signals from the nervous system to the periphery where the release of neural mediators affects immune responses and inflammation (9). Consequently, the nervous system is able to rapidly sense and regulate inflammation in peripheral tissues as well as restore immune homeostasis via the release of mediators acting locally on immune cells. In several chronic inflammatory diseases such as rheumatoid arthritis (RA), systemic lupus erythematosus, and IBD, the tone of the sympathetic nervous system (SNS) is also increased (10). This suggests that an autonomic nervous system imbalance with a dominant activation of the SNS and inadequate parasympathetic tone may have a key role in the pathogenesis of various immune related disorders including IBD (11).

## Neuroimmune Interaction in the Gut Wall

**Figure 1:**
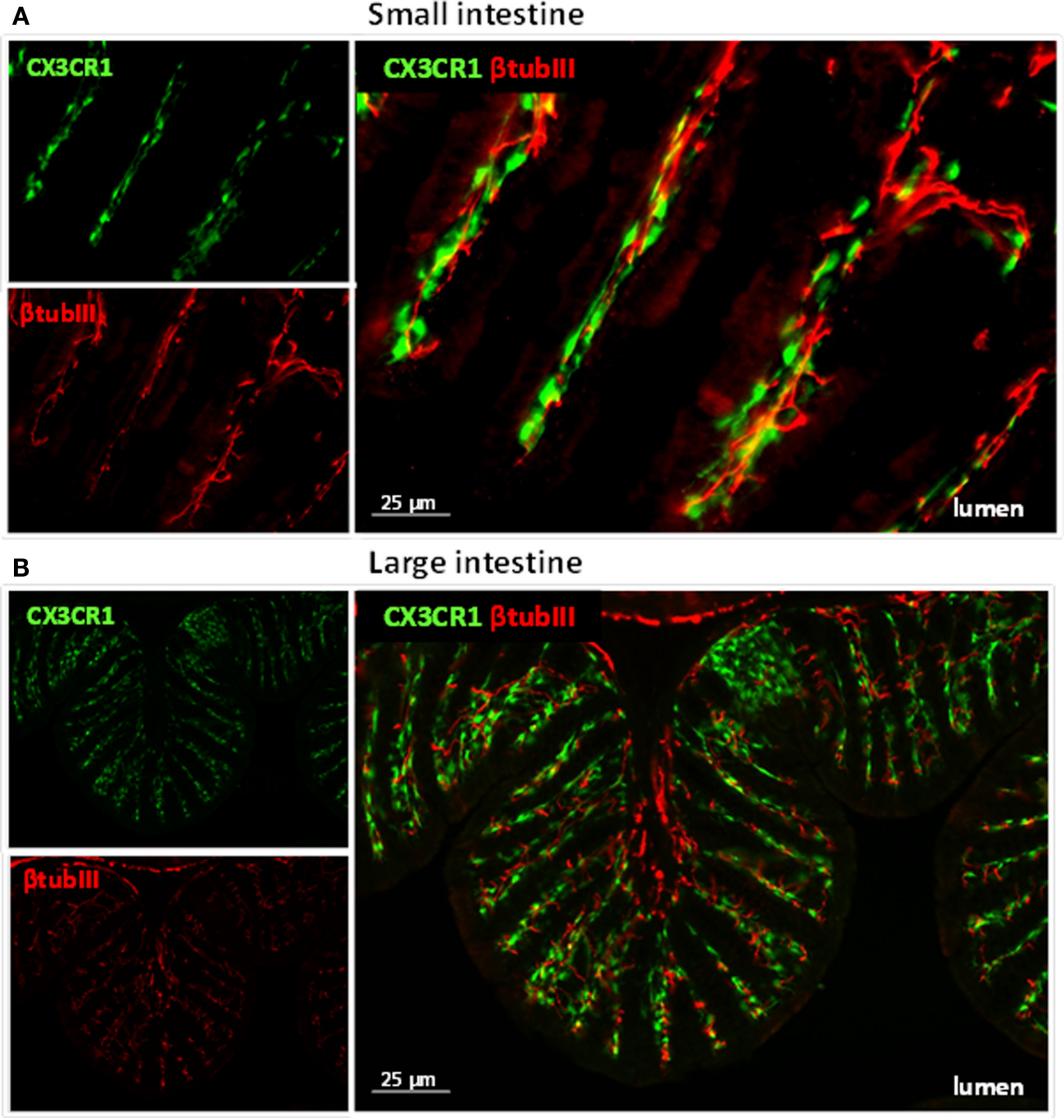
**Immune cells and neuronal fibers are in close proximity in the gastrointestinal tract**. Immunofluorescent picture of the ileum **(A)** and proximal colon **(B)** from CX3CR1^eGFP/WT^ mice showing in red neuronal fibers and in green CX3CR1^+^ macrophages. Neuronal fibers were visualized with a rabbit antitubulin III (red; Covance, 1:2000) followed by a donkey anti-rabbit Cy5 antibody (Jackson Immunoresearch). The green GFP signal (i.e., macrophages) highlights the CX3CR1^+^ macrophages.

The enteric nervous system (ENS), also known as the “Little brain of the gut,” forms a complex and independent nervous system within the GI tract (12). The ENS, together with the assistance of the extrinsic innervation, enables us to perceive the inner world of our intestine and its contents, to regulate motility and to digest nutrients (12) (Figures 1A,B).

The ENS consists of a network of hundreds of millions of neurons and glial cells clustered in small ganglia connected by nerve bundles organized in two major layers embedded in the gut wall, the myenteric *plexus* (or *Auerbach’s plexus*) and the submucosal *plexus* (or *Meissner’s plexus*) (13, 14).

**Figure 2:**
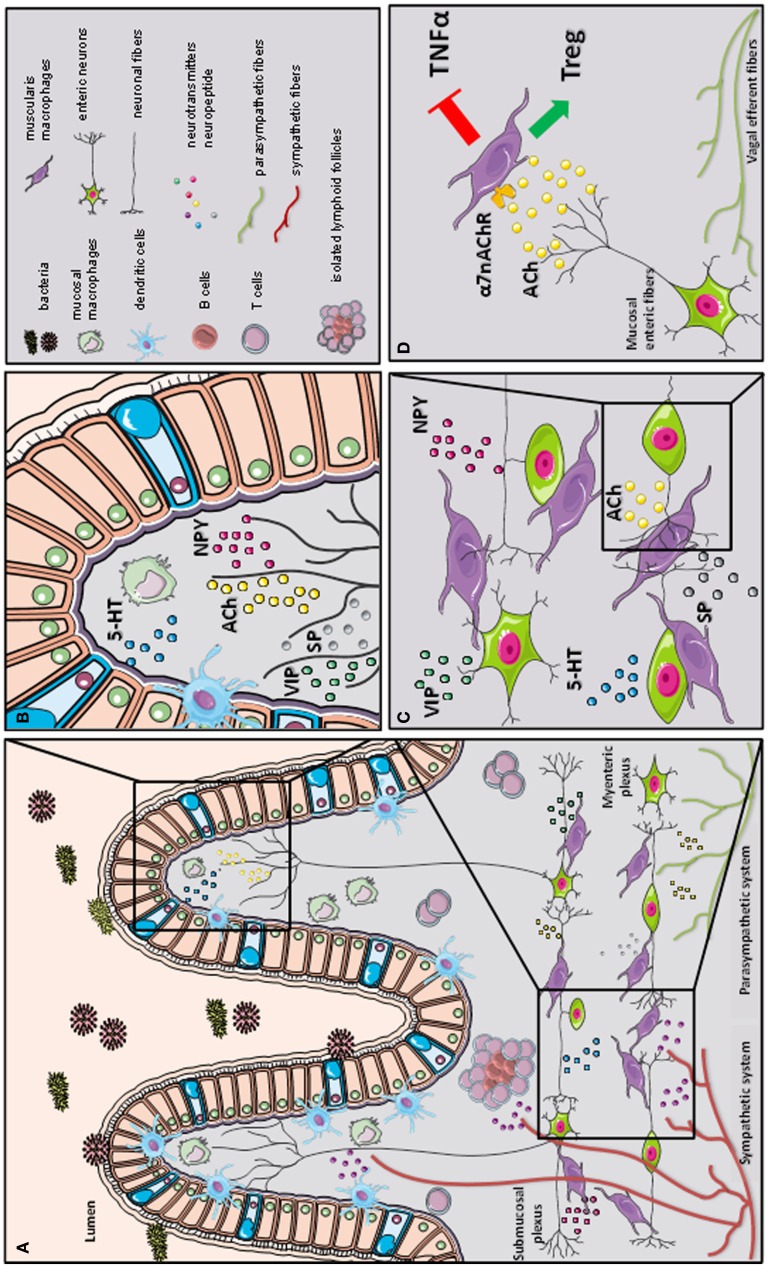
**A schematic representation of the cross-talk between the nervous and immune system in gastrointestinal tract**. The gastrointestinal tract is highly innervated via the autonomic nervous system (sympathetic and parasympathetic system) and enteric nervous system (via the myenteric and submucosal *plexus*). While the parasympathetic fibers (vagus nerve) extensively innervate the gut wall up to the myenteric *plexus*, the sympathetic fibers directly contact immune cells, secondary lymphoid organs (such as isolated lymphoid follicles), and enteric fibers in the submucosal/mucosal compartment **(A)**. Innate and adaptive immune cells, such as dendritic cells (DCs), macrophages (MFs), and T cells, located in the mucosal villi are affected by the presence of several immune-modulating neurotransmitters released by the enteric neural fibers, such as substance P (SP), vasoactive intestinal peptide (VIP), serotonin (5-HT), and neuropeptide y (NPY) **(B)**. In the myenteric *plexus*, a dense network of enteric neurons is present in close proximity to resident macrophages. The release of acetylcholine (ACh) and multiple of neuropeptides (SP, VIP, 5-HT, and NPY) condition the resident macrophages residing in the myenteric *plexus* **(C)**. During inflammation, vagal efferent fibers directly activate cholinergic enteric neurons in the myenteric *plexus*. The release of ACh triggers α7 subunit of the nicotinic acetylcholine receptor (α7nAChR) expressed on resident macrophages. The activation of α7 nAChR decreases tumor necrosis factor alpha (TNFα) release and favors regulatory T cells (Treg) conversion (our observation) leading to the control of intestinal inflammation and restoration of intestinal immune homeostasis **(D)**.

Functionally, the ENS resembles the central nervous system (CNS) as it uses similar sensory and motor neuronal fibers, information processing circuits “or interneurons” and releases a comparable set of neurotransmitters (Figure 2). The chemical neuromediators of the ENS were initially thought to be limited to neurotransmitters, such as acetylcholine (ACh) and serotonin, but, subsequently, purines, such as ATP, amino acids (γ-aminobutyric acid or glutamate), and peptides, such as vasoactive intestinal polypeptide (VIP) and neuropeptide Y (NPY), have been identified (15). More recently, nitric oxide (NO) has emerged as an important neurotransmitter in the ENS (16, 17). Overall, more than 20 candidate neurotransmitters have now been identified in ENS, and most neurons contain several of them (15). Growing evidence now supports the ground-breaking idea that these neurotransmitters can convey signals to immune cells and modulate their function (8). Indeed, many neurally derived molecules, such as ACh, serotonin and glutamate, have potent inhibitory effects on various immune cells including APCs or T cells (8, 18).

Besides the intrinsic innervation, the gut is extrinsically innervated by the autonomic nervous system composed of the sympathetic (SNS) and parasympathetic (PNS) system (Figure 2). Cell bodies of preganglionic sympathetic neurons are located in the CNS, between the first thoracic and third lumbar spinal cord segment, from where its axon connects to the postganglionic neurons where they send axons to the intestinal wall. On the contrary, parasympathetic preganglionic neurons are found directly in the brainstem were they form the vagus nerve (VN) that densely innervates the GI tract.

Sympathetic and parasympathetic fibers convey afferent information from the gut to the brain informing the CNS about the intestinal microenvironment (19). We have recently identified a direct activation of neurons (c-fos expression) in the dorsal motor nucleus of the VN in a mouse model of intestinal inflammation (20). This evidence strongly supports the idea that inflammatory stimuli can activate the autonomic nervous system and induce the release of immune-modulatory mediators in the periphery by nerve endings.

## Parasympathetic Nervous System as Modulator of Intestinal Immune Homeostasis

In line with the neuroimmune cross-talk hypothesis, the CNS, via the VN, is playing a crucial role in regulating immune response in the periphery through the cholinergic anti-inflammatory pathway (CAIP) (21). Tracey et al. originally reported that vagotomy significantly enhanced proinflammatory cytokine production and accelerated the development of septic shock, whereas electrical stimulation of the efferent VN prevented systemic inflammation and improved survival via the release of ACh in the spleen (22, 23). More recently, they also showed that in sepsis, the vagal anti-inflammatory reflex requires an intact splenic nerve (23) and alpha 7 nicotinic receptor (α7nAChR) expression on splenic MFs (24). Since the spleen is devoid of cholinergic innervation, this concept was lately challenged. However, this discrepancy was resolved by showing that upon vagus nerve stimulation (VNS), ACh released in the celiac mesenteric ganglia activates postsynaptic α7nAChR of the splenic nerve, leading to the release of norepinephrine in the spleen (25, 26). Surprisingly, norepinephrine induces the synthesis of ACh in a subpopulation of splenic memory T cell expressing choline acetyltransferase (ChAT), resulting into the anti-inflammatory effect of VNS (27). The discovery that activation of the VN has a potent anti-inflammatory effect implies that afferent vagal fibers will detect activation of the innate immune system in peripheral tissue and send an integrated efferent vagal input to that same site modulating the inflammatory response. Thus, the CAIP represents a new counter-regulatory mechanism to control the immune system and in contrast to circulating hormones or cytokines, provide a rapid local modulation of the immune response (27).

Early anatomical evidence highlights that efferent vagal nerve fibers extensively innervate the GI tract with a typical rostro-caudal gradient of vagal preganglionic innervation, with the highest density observed in the stomach followed by a subsequent decrease in the small bowel and colon (28). Therefore, it is not surprising that the vagal anti-inflammatory pathway plays a crucial role in the regulation of the intestinal immune response.

A few years later, we and others have extended the concept of the vagal anti-inflammatory pathway to the GI tract by showing the benefical effect of electrical VNS in a model of postoperative ileus (POI) (29) (Figure 2). POI is an immune-mediated condition evoked by surgical handling of the intestine in which the inflammation is primarily restricted to the intestinal muscular layer. In this model, electrical, nutritional and pharmacological activation of CAIP have been shown to prevent both surgery-induced inflammation and delayed GI transit (29, 30).

Similar to the spleen, we identified α7nAChR-expressing MFs residing in the *muscularis externa* as the final target of vagal anti-inflammatory pathway in the gut (Figure 2D) (31, 32). In addition, we have clearly verified that in POI, where inflammation only occurs in the *muscularis externa* the intestinal vagal anti-inflammatory effect is independent of the spleen and of ACh-producing T cells. As in the spleen, immune cells in the gut wall are indirectly modulated by the VN as vagal efferents solely synapse with cholinergic enteric neurons in the myenteric *plexus* (31). This implies that enteric neurons rather than vagal nerve endings interact with the intestinal immune system and release ACh.

Over the years, it is becoming increasingly clear that the microenvironment in the gut mucosa and submucosa determines the immune response to the initial exposure of luminal antigens. Given the potent anti-inflammatory effect of the cholinergic innervation, one might assume that the cholinergic tone in the submucosal compartment may have an important impact on mucosal immune homeostasis. The ENS forms a dense network of nerve fibers in close vicinity with intestinal immune cells, both in the submucosal (*lamina propria*) and *muscularis externa* compartment of the intestine (33). This could imply that vagal signals are amplified by the ENS inducing a substantial release of ACh in the intestinal microenvironment leading to modulation of the immune response. In line with this hypothesis, electrical and pharmacological activation of CAIP has been widely studied as a novel approach to treat IBD in several animal models. Reduced vagal cholinergic input induced by abdominal vagotomy results in an increased susceptibility to develop colitis with an elevated proinflammatory cytokines production following dextran sulfate sodium (DSS) administration (34). In line, nicotine or α7nAChR specific agonist treatments were protective in experimental colitis (34, 35). In addition, reduced mucosal levels of ACh in a murine model of depression were also associated with exacerbation of colitis (36, 37). However, the actual determinant of the increased susceptibility to colitis after vagotomy is still unknown. Interestingly, O’Mahony et al. recently showed that vagotomized mice have basal increase of activated nuclear factor (NF)-κB level in the gut and reduced splenic Tregs (38).

However, the crucial role of the α7nAChR revealed in models of sepsis and POI still remains ambiguous in colitis. α7nAChR^−/−^ mice had a higher severity of acute DSS-induced colitis which was in line with choline-chloride (α7nAChR specific agonist) treatment able to decrease inflammatory parameters in a model of depression-induced colitis (37). On the other hand, Snoek et al. described that treatment with specific α7nAChR agonists (AR-R17779 and GSK1345038A) reduced inflammation, such as NF-κB activity and cytokines, but these treatments did not improve the clinical signs of colitis (39), indicating that during colitis the receptor involved in vagal anti-inflammatory effect is still uncertain.

In accordance with the ambiguous effect of α7nAChR in experimental models of colitis, nicotine, an ACh nicotinic receptor agonist, has an opposite effect in the two main IBD forms: ulcerative colitis (UC) and Crohn’s disease (CD). Clinical observation indicates that UC patients experience more severe disease upon quitting smoking, while it improves again after returning to smoke (40, 41). In addition, a lower incidence of UC has been observed in smokers, while a negative effect of cigarette smoking has been observed in CD patients (41, 42). Smoking, namely, worsened symptoms compared to non-smokers in CD patients (43) due to increased influx of neutrophils into the intestinal mucosa (44, 45), suggesting that nicotine might affect colonic inflammation in IBD via CAIP. Galitovskiy et al. proposed a possible experimental explanation for the dichotomous effect of nicotine in colitis. In this study, the authors showed that nicotine attenuated oxazolone colitis, resembling UC (Th2-induced inflammation), increasing colonic regulatory T cells and reducing Th17 cells. On the contrary, nicotine exacerbated trinitrobenzene sulfonic acid (TNBS-) induce colitis, resembling CD (Th1-induced inflammation), by increasing colitogenic Th17 cells (46). These findings suggest that nicotine might influence the inflammation according to the type of immune response partially explaining the controversial effect of smoking observed in UC and CD patients.

Various studies have tried to correlate autonomic dysfunction, such as alteration of the vagal tone, with clinical outcome in IBD patients (47, 48). Bonaz and colleagues reported negative correlation between low vagal tone and increase plasma levels of tumor necrosis factor alpha (TNF-α), suggesting that the CAIP may be altered in these patients. However, a clear correlation between IBD and vagal tone has still not been convincingly verified. Recently, Clarençon et al. described the first attempt of VNS in a patient with CD. The patient subjected to long-term low frequency VNS, showed significant improvement with reduction of both clinical disease activity index and endoscopic remission. This beneficial effect was correlated to an increased parasympathetic tone (49). Even though this report is interesting in this patient proposing a therapeutic role for the VNS in IBD, results should be taken with caution considering the size of the study and the fact that placebo effect could not be ruled out using this experimental approach.

Currently, electrical VNS is explored as therapeutic treatment in patients affected by chronic inflammation (50). Ongoing clinical trials are investigating the possible beneficial effect of VNS in patients with RA, POI, and CD (NCT01552941, NCT01569503, and NCT01572155). Additional preclinical and clinical data will hopefully clarify whether the CAIP will be an alternative therapeutic approach to treat intestinal inflammatory diseases.

## Sympathetic Nervous System

Besides the PNS, the GI tract is extensively innervated by the sympathetic nervous system (SNS) mostly involved in the modulation of blood flow, secretion, and motility.

Sympathetic fibers mainly innervate the myenteric and submucosal *plexus* as well as the mucosal layer (51). Of note, on the contrary to the PNS, sympathetic fibers have been found in direct contact with immune cells residing in gut-associated lymphoid tissues including Peyer’s patches and MLN (52, 53). Anatomical studies have undoubtedly shown large amounts of noradrenergic fibers both into the dome region of the follicles where fibers are in direct contact with lymphoid cells and in the *lamina propria* where fibers are mainly associated with blood vessels (52). Interestingly, various innate and adaptive immune cells express receptors for the typical sympathetic neurotransmitters, including noradrenaline (NA) and adrenaline (A), supporting the idea that also the SNS may regulate immune response and inflammation in peripheral tissues including the GI tract (25, 54, 55). Catecholamines bind a large family of adrenergic receptors. These are G-couple receptors composed of different subunits: three α1 (A, B, and D), three α2 (A–C), and three β (β1–β3) receptor subtypes. Interestingly, adrenergic receptors have different threshold of activation depending on the cathecholamine concentration: high concentrations activate β-adrenoreceptors, subsequently increasing cAMP levels, whereas low concentrations activate preferentially α-adrenoreceptors leading to decreased cAMP levels (56). This difference may explain the controversial results obtained in experimental and clinical studies investigating the pro- and anti-inflammatory role of sympathetic innervation during local tissue inflammation. It is important to mention that catecholamines can also be produced and released by various immune cells, such as T- and B-cells (57). Interestingly, production of catecholamines after immune cell activation has been proposed as an autocrine loop involved in the regulation of inflammation (58).

Together with anatomical proximity of noradrenergic fibers to various immune cells (59), also a direct functional interaction has been proven on DCs. Recent studies, demonstrated direct effects of NA on DC migration, antigen uptake, cytokine production and T cell polarization via the intracellular signaling pathways PI3K and ERK1/2 (60–63). Additionally, adrenergic fibers also affect T cell polarization repressing Th1 polarization, while favoring Th2 cell induction (64, 65), suggesting possible effect of NA in the skewing of T cell responses.

The reciprocal cross-talk between the sympathetic fibers and immune system has been extensively studied also in experimental models of intestinal inflammation.

In order to study the role of sympathetic immune-regulation during inflammation, chemical sympathectomy has been performed using 6-hydroxydopamine (6-OHDA) treatment resulting into depletion of NA in the peripheral nerve terminals. In rats, 6-OHDA treatment revealed an alteration in migration and accumulation of lymphocytes (B and T cells) in the gut-associated lymphoid organs during inflammation (66, 67). In more detail, sympathetic denervation decreased inflammation in acute DSS- and TNBS-induced colitis (68, 69). On the contrary, Straub and colleagues showed in two different chronic models of colitis (i.e., chronic DSS-induced colitis and IL10-deficient mice) that chemical sympathectomy significantly exacerbated disease, suggesting that catecholamines may play a favorable effect in the chronic phase of inflammation by promoting tissue repair (70). These controversial results might be explained by a dual role of these fibers in which the SNS confers proinflammatory effects at the beginning of colonic inflammation, while it exerts anti-inflammatory effects in the chronic phase of inflammation. Of note, similar results have been recently described in a murine model of rheumatoid arthritis, another chronic model of inflammation. Indeed, mice that underwent early sympathectomy showed improvement of arthritis scores while animals subjected to late sympathectomy had significantly increased arthritis scores compared with control mice (71).

In addition to sympathectomy, several studies have attempted to mimic the effect of the SNS during colitis by pharmacological activation of adrenergic receptors. Treatment with a β_3_-AR agonist was shown to be beneficial as it reduced the severity of TNBS-induced colitis in rat (72). However, this anti-inflammatory effect of the agonist might be indirect, since it was associated with cholinergic-mediated contractions of the colon known to improve mucosal healing (73). However, in another study activation of β_3_-ARs was also able to reduce colonic cytokines release, further supporting its anti-inflammatory effect in colitis (51). Additionally, during TNBS-induced colitis, mice treated with α2-adrenoceptor antagonist, RX821002, showed a reduced expression of colonic proinflammatory genes (TNF-α and IL-1β) (74). Overall, preclinical data clearly indicate the immunomodulatory effect of the SNS in experimental models of colitis. However, the effect of the SNS is still controversial with studies reporting both pro and anti-inflammatory effects, depending on the preclinical model used and on the disease stage assessed (69).

As previously mentioned, accumulating evidence suggests that abnormalities in the neural autonomic profile may be an aggravating factors in the pathogenesis of IBD (50). Of note, enzymes involved in the synthesis of NA are reduced in patients affected by both UC and CD even though lower level of NA has been only detected in CD patients (75, 76). Furthermore, a reduction of sympathetic nerve fibers has been observed in biopsies from CD patients, while sympathetic fibers are increased in tissue of UC patients (77, 78).

The idea that SNS dysfunction may have an impact on IBD has been recently tested with promising results in a clinical trial in UC patients. In this study, treatment with the α_2_-AR-agonist clonidine, induced normalization of the SNS activity and significantly improved disease severity in patients with active UC (79).

Although the involvement of the SNS in the inflammatory response has been extensively proven, further studies should be conducted to better understand how modulation of the SNS may enter in the therapeutic armamentarium of IBD.

## The Peptidergic Pathway: Neuropeptides

Neuropeptides are neuronal signaling peptides involved in a wide range of neuronal functions. Often neuropeptides are coreleased with neurotransmitters complicating studies evaluating their specific effects. Neuropeptides modulate neuronal communication by acting on cell surface receptors. Interestingly, various subtypes of neuropeptide receptors are also expressed on immune cells suggesting possible influence of these molecules on the immune system (Table 1). This idea is supported by clinical and experimental observations obtained in IBD patients and animal models of colitis that are discussed below.

**Table 1:** **Expression and effect of selected neurotransmitters during intestinal inflammation**.

Neurotrasmitter	Main source	Receptors	Target cells	Experimental evidence	Clinical evidence
**Catecholamines**					
Noradrenaline–adrenaline	Sympathetic fibers, T and B cells (59)	β-adrenoreceptors (58) α-adrenoreceptors (58)	DCs, T cells (25, 56, 57)	Sympathetic denervation improves DSS, TNBS (78, 79) Sympathetic denervation worsens IL10^−/−^ and chronic DSS (77, 80, 81) β_3_-AR agonist improves TNBS (82) α2-Adrenoceptor antagonist improves TNBS (83)	Active UC patients typically have increased SNS activity (71–73), it is decreased in CD patients (74) α2-AR-agonist improve severity of UC in human (71)
Acetylcholine	Parasympathetic fibers Cholinergic enteric neurons T and B cells (23–27)	α7 nicotinic acetylcholine receptor (26)	Macrophages (22, 26, 31)	Vagotomy worsens DSS (34) Choline-chloride improves depression-induced colitis (37) Nicotine improves oxazolone colitis (46) Nicotine worsens TNBS (46) α7nAChR^−/−^ mice had a higher severity of acute DSS-induced colitis (39)	VNS improves severity CD patient (49)
**Neuropeptides**					
Vasoactive intestinal peptide (VIP)	Enteric neurons (81)	VPAC1, VPAC2 (80)	Smooth muscle, T cells, DCs, macrophages (80–82, 84, 85)	Immunomodulatory effects (86–92) VIP treatment ameliorates TNBS colitis (93)	Limited clinical evidence: both ↑ and ↓ of VIP level observed in IBD (94–96)
Neuropeptide Y (NPY)	Central and peripheral nervous system (97) Immune cells (105)	Y1, Y2, Y3, Y4, Y5, and Y6 (GPCRs) (98, 99)	Innate and adaptive immune cells (monocytes, lymphocytes, and granulocytes) (100–103)	Proinflammatory effect: shown in mouse and rat models of DSS Induced colitis (106–108) and TNBS-induced colitis (109)	IBD patients: no change in NPY plasma levels (104)
Calcitonin gene-related peptide (CGRP)	α-CGRP: central and peripheral nervous system β-CGRP: gut, pituitary gland, and immune system (119–121)	Calcitonin receptor-like receptor (CRLR) (110)	TRPV1 and CGRP are colocalized on peripheral neurons and on immune cells, such as MFs and DCs (111–115)	Anti-inflammatory effect Shown in mouse and rat models of DSS-induced colitis (122) and TNBS-induced colitis (123)	UC and CD patients: ↓ CGRP^+^ cells in the intestinal *muscularis* layer (116–118)
Substance P (SP)	Central and peripheral nervous system (124–126) Innate immune cells: monocytes, MFs, eosinophils and lymphocytes (133–136)	Neurokinins-1 (NK-1), NK-2, and NK-3 (GPCRs) (127–130)	Enteric neurons, smooth muscle, endothelial cells, immune effectors, and mucosal epithelial cells (127–130)	Contradictory results Proinflammatory effect: shown in mouse and rat models of DSS colitis (137) and TNBS colitis (138) Anti-inflammatory effect: role in mucosal healing shown in mouse models of DSS and TNBS colitis (140, 141)	UC patients ↑ SP in rectum and colon (77, 131, 132) SP in severe inflammatory colon lesions (139) CD patients: decreased (131), unchanged (142), and increased (77) SP levels
Serotonin	Enterochromaffin cells, enteric neurons (143, 144)	5-HT receptors (143, 144)	Enteric neurons, immune cells (145, 146)	↓ Serotonin (genetic and pharmacological) ameliorates DSS and DNBS colitis (147); ↑ serotonin worsens colitis in IL10^−/−^ mice (148)	No clinical evidence available

## Vasoactive Intestinal Peptide

Vasoactive intestinal peptide is a 28-amino acid neuropeptide that is widely expressed in different tissues, including intestine, central and peripheral nervous system, pancreas, and lung (80). In the intestine, VIP is mainly produced by enteric neurons in the myenteric and submucosal *plexus* and regulates both intestinal motility and chloride secretion (81, 82, 84). In addition, VIP has emerged as a potent anti-inflammatory peptide affecting both innate and adaptive immune responses. Due to its anti-inflammatory properties, the therapeutic potential of VIP in the treatment of inflammatory disorders, such as IBD, has been extensively investigated. In the intestine, the receptors for VIP (VPAC1 and VPAC2), are mainly expressed by smooth muscle cells and immune cells including T cells, DCs, and MFs, suggesting a possible immune-modulatory effect of VIP (80, 83, 85). The *in vitro* anti-inflammatory properties of VIP on myeloid cells are well documented and highly relevant for IBD. Indeed, one of the proposed pathogenic drivers in IBD is the loss of immune tolerance against harmless antigens leading to chronic production of inflammatory mediators by MFs and enhanced Th1/Th17 polarization by inflammatory DCs (149, 150). Interestingly, VIP inhibits inflammatory MFs through inhibition of NF-κB activation leading to reduced production of proinflammatory cytokines (TNFα, IL-6, and IL12p40) and enhanced production of the tolerogenic cytokine IL-10 (151–155). Moreover, VIP treatment reduces expression of toll-like receptor 4 (TLR4) in MFs rendering them less responsive to lipopolysaccharide (LPS) (86, 87). In addition, DCs acquire an anti-inflammatory phenotype upon VIP treatment. VIP-induced activation of VPAC1 in bone marrow-derived DCs induces a tolerogenic phenotype with low levels of costimulatory molecules CD80, CD86, and CD40, and high levels of the anti-inflammatory cytokine IL-10 (88, 89). These VIP-conditioned DCs induce polarization of naïve CD4^+^ T cells into CTLA4^pos^ IL-10 secreting Tregs. In addition to its effects on innate immune cells, VIP also acts directly on T cells to promote Th2 differentiation through activation of VPAC2 (90, 91). Notably, during Th2 differentiation both VIP and VPAC2 are upregulated in T cells, suggesting that VIP-VPAC2 participates in a Th2 autoregulatory loop (92). It has indeed been demonstrated that VIP supports survival of Th2, but not Th1 cells (90). Taken together, by acting on both the innate and adaptive arm, VIP actively counteracts Th1 responses. These findings highlight VIP as an attractive therapeutic candidate for the treatment autoimmune disorders with a typical Th1 profile, including CD. The therapeutic potential of VIP in CD is indeed supported by the beneficial effects of VIP in TNBS-induced colitis, an experimental model of CD (156). Administration of VIP induces a remarkable amelioration of TNBS-induced colitis through activation of VPAC1. Reduced disease severity correlates with lower levels of proinflammatory chemokines and cytokines, inhibition of Th1 responses and induction of a Th2 immune response (156, 157). Although VIP showed very profound effects in this study, it should be noted that other studies could not confirm the beneficial effects of VIP in TNBS-induced colitis (158). Moreover, inhibition or genetic deletion of VIP does not exacerbate colitis, but rather induces resistance to both TNBS- and DSS-induced colitis (93, 159). These findings make the interpretation of the preclinical therapeutic evidence of VIP in colitis difficult and argue for further fundamental research to better understand the promiscuous role of VIP in intestinal physiology and pathology. Also from a clinical point of view, the role of VIP in IBD is far from clear. Although there are some reports showing hypertrophic VIPergic nerves and increased VIP levels in rectal biopsies of patients with IBD, other reports demonstrate no increase or even decreased levels of VIP (94–96). In summary, although VIP exerts potent anti-inflammatory effects *in vitro*, there is currently not enough preclinical and clinical evidence to support translation of VIP treatment in IBD to the clinic.

## Neuropeptide Y

Neuropeptide Y is a 36-amino acid peptide and is considered as one of the most abundant peptides in the central and peripheral nervous system (97). NPY is highly conserved among species and in mammals its effects are mediated trough binding of six different G-coupled receptor subtypes (Y1, Y2, Y3, Y4, Y5, and Y6) (98, 99). In the CNS, NPY is mainly present in the hypothalamus and is involved in modulating anxiety, appetite, blood pressure, and nociception (160–162). On the other hand in the periphery, it is mainly expressed in sympathetic nerves where it is colocalized and coreleased with NA. Within the gut, enteric neurons of the *myenteric plexus* and *submucosal plexus* are the major source of NPY (163). The biological effects of NPY on the GI system are of inhibitory nature and include effects on pancreatic and GI secretion, blood pressure, and GI motility as well as modulation of intestinal inflammation with direct interaction with the immune system (164). NPY can affect both the cells of the innate and adaptive immune system as it modulates neutrophil chemotaxis, granulocyte oxidative burst, and NO production, T helper cell differentiation, natural killer cell activity, suppression of lymphocyte proliferation and activation of APCs (165–168). Interestingly, next to nerve-derived NPY, immune cells themselves are able to express NPY enabling them to modulate the immune cell function in a paracrine or autocrine manner (105). The expression of several Y receptor subtypes have been reported on immune cells (100–103). As Y1 receptor is the most abundant receptor, it has been intensively investigated and appears to be present in each immune cell investigated so far (100). Although less information has been gathered about the expression of other Y receptors, human neutrophils express Y1, Y2, Y4, and Y5, with Y4 being the most abundant (169). Furthermore, Y1, Y2, and Y5 expression was also demonstrated in mouse MFs and rat granulocytes (103, 170). NPY can exert pro- or anti-inflammatory effects, depending on which receptor is activated and on which immune cell. In particular, NPY levels were shown to be increased in both DSS-induced colitis (171) and TNBS-induced colitis (109). Further evidence for the role of NPY in promoting inflammation in the gut is provided by the fact that NPY knockout (KO) mice are resistant to the induction of DSS colitis (106, 107). Similar results were obtained by using an NPY antisense oligodeoxynucleotide in rats (171). Receptor Y1 seems to mediate the proinflammatory effect of NPY as KO or antagonism of the receptor results into a comparable attenuation of inflammation (108). In addition, receptor Y1-deficient mice have impaired APC function and consequently a decreased number of effector T cells, as well as a decreased MF production of TNF-α and IL-12, explaining the observed protective phenotype in experimental colitis (102). During gut inflammation, there is a considerable amount of cross-talk between NPY and TNF-α. This was shown by the decreased production of TNF*-*α by enteric neuronal cells from NPY-deficient mice. Conversely, block of TNF-α causes a reduction in colonic NPY expression (172). Additionally, NPY also enhances nNOS, which is associated with oxidative stress, in a murine model of DSS colitis (106). Although NPY plasma level is not altered in IBD patients (104), targeting NPY or its receptors might be an interesting therapeutic approach for treating IBD.

## Calcitonin Gene-Related Peptide

Calcitonin gene-related peptide (CGRP) is a 37-amino acid neuropeptide that exists in two isoforms, α-CGRP and β-CGRP. These isoforms are encoded by two different, but closely related genes. α-CGRP is mainly produced in the central and peripheral nervous system, whereas β-CGRP is primarily produced in the gut, pituitary gland and by immune cells. The biological actions of both isoforms are largely overlapping (119–121). Although CGRP participates in development and maintenance of pain, it has been also described as a potent regulator of inflammatory responses (173). In addition in the GI tract, CGRP participates in the regulation of gastric acid secretion and intestinal motility (174, 175). Release of CGRP by nerve endings and immune cells is induced by activation of the transient receptor potential vanilloid 1 (TRPV1). TRPV1 and CGRP are colocalized on peripheral neurons as well as on immune cells, such as MFs and DCs (111–115). Whereas the anti-inflammatory effect of CGRP on LPS-induced inflammation is well described, its role in intestinal inflammation still needs to be fully elucidated. Up to now numerous studies have pharmacologically investigated the role of CGRP in experimental models of colitis. The effect of systemic administration of CGRP and its antagonist, hCGRP, has been tested in a rat TNBS model. Intravenous administration of CGRP protected the colonic mucosa against TNBS in both the early and late phases of acute colitis, while hCGRP exacerbated TNBS-induced inflammation (123). On the other hand, the contribution of TRPV1 in gut inflammation is still controversial, as it was shown that disease severity was reduced in TRPV1 KO mice in model of DSS colitis, suggesting that TRPV1 activation may enhances inflammation (176). On the contrary, acute stimulation of sensory neurons by capsaicin, a known TRPV1 agonist, ameliorated disease symptoms in TNB-colitis in rats (177). This could be due to the corelease of other neuropeptides from sensory TRPV1^+^ fibers, such as substance P, which could promote a more proinflammatory milieu (122). Recently, CGRP expression has also been reported in TRPM8 expressing (temperature-sensitive TRP channels for cold sensation) mucosal fibers. In line, TRPM8-dependent CGRP release have been shown in the colon upon DSS exposure (122). In the same study, genetic deletion of TRPM8 increased the susceptibility of mice to acute colitis. This was correlated with an increase in CGRP levels in mucosal fibers suggesting that TRPM8-CGRP signaling may be involved in dampening intestinal inflammation (122).

Considering the possible participation of CGRP in the pathogenesis of IBD many studies have attempted to gather clinical data in both UC and CD patients. However, while early studies showed a decrease of CGRP positive cells in the intestinal *muscularis* layer of UC and CD patients (116–118), a more recent report did not confirmed lower CGRP level in tissue from patients during moderate and severe UC (178).

## Substance P

Substance P is a neuropeptide which is composed of 11-amino acids (179) and is widely expressed in the brain and periphery (124) including the GI tract where it is mainly expressed by neurons of the *myenteric* and *submucosal plexuses*, as well as intrinsic and extrinsic sensory neurons (125, 126). Interestingly, SP is also expressed by a variety of innate immune cells, such as monocytes (133), MFs (136), eosinophils (134), and lymphocytes (135). SP binds specific G protein-coupled receptors named neurokinins-1 (NK-1), NK-2, and NK-3. Within these three receptors, NK-1 has the highest affinity for SP and is abundantly expressed throughout the GI tract by different kind of cells: enteric neurons, smooth muscle, endothelial cells, immune effectors, and mucosal epithelial cells (127–130). Several studies have correlated SP-NK-1 signaling with intestinal inflammation. In a model of TNBS colitis, mice lacking neutral endopeptidase (NEP), an enzyme that degrades SP in the extracellular fluid, displayed exacerbated inflammation (138). Accordingly, the use of an NK-1 antagonist improved the severity of colitis in different experimental models (137, 138). Binding of SP to its receptors leads to the activation of mitogen-activated protein (MAP) kinase, protein kinase C (PKC), and NF-kB pathways leading to production of proinflammatory cytokines, such as IL-1b, IL-6, IL-8, and TNFα (180–183). Although these findings suggest a proinflammatory effect of SP in intestinal inflammation, studies have emerged that highlight a role for SP in mucosal healing and thus propose a beneficial effect of SP in colitis. Castagliuolo I et al. described the development of DSS and TNBS-induced colitis in NK-1 receptors deficient mice, and showed that there was an increase in severity of colitis as well as an increased mortality in these mice (140). The protective effect of NK-1 is associated with the transactivation of epidermal growth factor receptor (EGF-R), which in turn leads to cell proliferation in the colon (140, 141). Moreover, SP has been described to trigger cell proliferation in a multitude of cell types, such as T cells, smooth muscle cells (184). Although clinical evidence suggests a role for SP in the pathophysiology of IBD in patients, the exact role of SP in intestinal inflammation needs to be further elucidated. Several studies investigated levels of SP in the serum and locally in the gut. Increased levels of SP have been observed in the rectum and colon of UC patients, and were correlated with disease activity (77, 131, 132). On the other hand, another study showed that SP containing nerves increased in hypervascularized lesions, while they decreased in severe inflammatory lesions in the colon of UC patients (139). Contradictory results have also emerged in studies investigating SP in CD patients. While some reported decreased levels of SP in the mucosa in CD patients (131), others showed that there is no difference (142) or even an increase in SP levels (77). These controversial results might be explained by the variation in methodology, tissues used and different stages of the disease (185–187). Additionally, changes in NK-1R expression were investigated in IBD patients. An increased number of NK-1R positive lymphoid cells was observed and additionally also increased NK-1R mRNA expression in inflamed mucosa was reported. Furthermore, an altered pattern of epithelial NK1-R expression was found in UC, while in CD an increased expression was reported in the *myenteric plexus* (125). Clearly, further studies are needed in order to clarify the interaction between SP and the immune system and its possible involvement in intestinal inflammation.

## Serotonin

Although serotonin or 5-hydroxytryptamine (5-HT) is well known as a neurotransmitter of the CNS, the majority of serotonin in the human body is produced in the GI tract (143). Intestinal 5-HT is mainly produced by enterochromaffin cells (ECs) and enteric neurons of the myenteric plexus (144, 188). In both cells types, biosynthesis of 5-HT depends on the conversion of dietary l-tryptophan to 5-hydroxytryptophan by tryptophan hydroxylase (TPH). Synthesis of 5-HT in the intestine is performed by two different types of TPH, namely TPH1 and TPH2 (145). The bulk of 5-HT is produced by ECs and depends on TPH1. In contrast, enteric neurons only have a minor contribution to total 5-HT levels in the intestine and rely on TPH2 for the production of 5-HT. The distinct regulation of 5-HT synthesis in these two compartments also has important functional implications. Whereas myenteric production of 5-HT by TPH2 is essential for normal motility, mucosal 5-HT synthesis by TPH1 in ECs influences intestinal inflammation (145, 147). Indeed, depletion of mucosal 5-HT via genetic deletion ameliorates both DSS- and DNBS-induced colitis in mice. This effect is eliminated when TPH1 knockout mice are replenished with 5-HT (147). In line, mice that develop spontaneous colitis due to IL-10 deficiency have increased disease severity when the actions of 5-HT are amplified by genetic deletion of the serotonin transporter SERT (148). Enterocytes of SERT knockout mice are unable to remove 5-HT from the extracellular space leading to enhanced 5-HT effects. Taken together, these data clearly show that mucosal 5-HT has profound proinflammatory effects and can exacerbate development of colitis. Hence, selective inhibition of mucosal 5-HT levels might be an interesting therapeutic option for the treatment of IBD. In a recent study, the therapeutic potential of selective TPH1 inhibition has been validated. Indeed, oral administration of telotristat etiprate, a potent TPH1 inhibitor, effectively reduced disease severity and intestinal inflammation in mice with TNBS-induced colitis (146). Of note, telotristat etiprate did not cross the blood–brain barrier nor did it affect serotonin levels in myenteric neurons (189, 190). This is important to ensure the safety of the treatment and to avoid side effects due to depletion of 5-HT in the CNS and ENS. Another way to reduce the proinflammatory effects of 5-HT is by inhibition of 5-HT receptors that are selectively expressed on intestinal immune cells. Although the mechanisms by which 5-HT exerts its proinflammatory actions is not completely understood, it was recently shown that DCs isolated from TPH1 deficient mice produce less IL-12 compared to wild-type DCs (191). Treatment of these DCs with 5-HT led to restoration of IL-12 production, indicating that 5-HT can polarize DCs to become proinflammatory. Hence, selective pharmacological inhibition of 5-HT might be an interesting therapeutic approach since the proinflammatory actions of 5-HT on intestinal DCs could be reduced. Unfortunately, the 5-HT receptors that are commonly found on intestinal DCs are also involved in other processes in both CNS and ENS. Accordingly, inhibition of 5-HT receptor signaling has a relatively poor therapeutic potential given the severe side effects, including behavioral and motility problems. In summary, reduction of mucosal 5-HT through selective inhibition of TPH1 likely holds most promise for future treatment of inflammatory intestinal disorders. However, further research is needed to better define the cellular players involved in the proinflammatory actions of 5-HT.

## Conclusion and Perspective

An intricate network of immune and non-immune cells and their mediators function in unison to protect us from toxic elements and infectious microbial diseases that are encountered in the intestinal lumen. This network operates efficiently by use of a single cell epithelium layer, fortified by adjacent cells and lymphoid tissues that protect its integrity. On occasion alterations of the steady state due to genetic background and/or environmental microbes result in inflammatory diseases or infections including development of IBD. Thus, intestinal immune homeostasis is finely regulated by several redundant strategies to counteract excessive and unnecessary immune responses. Lately, an intimate bidirectional interaction between the nervous and immune system in the gut has been increasingly demonstrated. This pathway is a hard-wired connection between the immune and nervous system closely interacting to regulate inflammation. Based on our and others’ findings, we can now conclude that intestinal neuronal circuits modulate the immune response directly into the gut wall. Therefore, we hypothesize that there may be two stages of neural modulation in the gut: in case of subtle and localized intestinal inflammation, the local innervation involving the ENS will be activated, while in more systemic inflammatory responses, such as in severe colitis, the autonomic innervation will come into play and modulate the immune response even in distant organs, such as the spleen or the bone marrow.

In conclusion, studying the immune-modulatory properties of nervous systems as well as endogenous neuropeptides will be a fundamental challenge for the next years. Although further fundamental understanding of their role in specific immune disorders is required, it is evident that neuroimmunomodulatory therapies hold great promise, as evidenced by the ongoing clinical trials evaluating the effect of CAIP in autoimmune disorders. Further understanding of the neuronal circuits and receptors involved will likely support the development and use of specific receptor agonists and antagonists in the treatment of different neuroimmune pathologies. These new insights will not only affect our understanding of GI function, but might help to elucidate the complex interactions in other organs and systems. Unraveling of the mechanisms by which the autonomic and intrinsic innervation may reinstall intestinal immune homeostasis will, therefore, have a major impact on the therapeutic approach of many so far untreatable disorders, such as IBD.

## Conflict of Interest Statement

The authors declare that the research was conducted in the absence of any commercial or financial relationships that could be construed as a potential conflict of interest.
